# Evaluation of left atrial function and mechanical dispersion in breast cancer patients after chemotherapy

**DOI:** 10.1002/clc.23813

**Published:** 2022-03-16

**Authors:** Na Chen, Ansheng Liu, Siyao Sun, Hong Wei, Qiaobing Sun, Zhijuan Shang, Yinghui Sun, Tingting Fu, Hongjiang Wang, Yunlong Xia, Lanqi Hua, David H. Hsi, Tao Cong

**Affiliations:** ^1^ Department of Cardiovascular Ultrasound First Affiliated Hospital of Dalian Medical University Dalian Liaoning People's Republic of China; ^2^ Department of Breast Surgery First Affiliated Hospital of Dalian Medical University Dalian Liaoning People's Republic of China; ^3^ Department of Cardiovascular Ultrasound Massachusetts General Hospital Cardiac Ultrasound Lab Boston Massachusetts USA; ^4^ Department of Cardiology Stamford Hospital Stamford Connecticut USA

**Keywords:** breast cancer, cardiotoxicity, chemotherapy, echocardiography, left atrial function, left atrial mechanical dispersion

## Abstract

**Background:**

Left atrial (LA) function and mechanical dispersion changes in breast cancer patients treated with chemotherapy remain unclear.

**Hypothesis:**

LA function and LA mechanical dispersion in breast cancer patients would be impaired after chemotherapy.

**Methods:**

This single‐center retrospective study included 91 consecutive breast cancer patients treated with chemotherapy and 30 controls. Patients were examined by echocardiography three times at intervals. Conventional parameters, left ventricular strain, LA strain, and LA mechanical dispersion were evaluated and compared.

**Results:**

LA strain during reservoir phase (LASr), conduit phase (LAScd), and contraction phase (LASct) all decreased markedly after chemotherapy and were lower than those of the controls (all *p* < .01). The standard deviation of time to peak positive strain during LA reservoir phase corrected by R‐R interval (LA SD‐TPSr) was significantly increased after chemotherapy and was higher than that of the controls (*p* < .001). The change of LA function was expressed as Δ. Multivariate linear regression analyses showed that LAVIp (0.399, 95% confidence interval [CI]: 0.610, 1.756, *p* = .000) was independently associated with ΔLASr, LAPEF (−0.325, 95% CI: −45.123, −10.676, *p* = .002) and age (0.227, 95% CI: 0.021, 0.350, *p* = .027) were independently associated with ΔLAScd, and LAVImax (0.341, 95% CI: 0.192, 0.723, *p* = .001) was independently associated with ΔLASct. LAVImax (0.505, 95% CI: 0.000, 0.001, *p* = .039) and mitral E (−0.256, 95% CI: 0.000, 0.000, *p* = .024）were independently associated with ΔLA SD‐TPSr.

**Conclusions:**

Mechanical function of LA declined after chemotherapy in breast cancer patients. With the decrease of LA mechanical function, LA mechanical dispersion assessed by two‐dimensional speckle‐tracking echocardiography increased significantly, and its clinical value needs to be further studied.

## 1 INTRODUCTION

Breast cancer remains the leading cause of mortality in women. In the United States, breast cancer affects nearly 3.32 million women.^1^ Currently, advances in breast cancer treatment have led to improved survival in these patients. However, treatment can result in cancer therapeutic‐related cardiac dysfunction (CTRCD) due to myocardial toxicity.^2^, ^3^ CTRCD affects a substantial portion of patients who undergo chemotherapy. Conventionally, parameters of the left ventricle (LV), such as left ventricular ejection fraction (LVEF) and LV global longitudinal strain (GLS), have been broadly used as diagnostic criteria for myocardial damage.^4^ Most of the previous studies have focused on LV dysfunction after chemotherapy. However, left atrial (LA) function has recently been identified as a potential indicator of cardiac dysfunction and arrhythmias due to cancer treatments.^5^ In addition, controversies remain regarding changes in LA strain in breast cancer patients after chemotherapy. Ana Teresa Timóteo et al. suggested that no significant change in LA strain in breast cancer patients was observed after chemotherapy.^6^ In contrast, Hyukjin Park et al. suggested that a significant decline in LA strain developed after chemotherapy for breast cancer.^7^ The respective changes in LASr, LAScd, and LASct in these patients is still a matter of debate. LA mechanical dispersion is a parameter related to arrhythmia, especially atrial fibrillation (AF).^8^ However, data on LA mechanical dispersion in breast cancer patients after chemotherapy remain limited. Echocardiography is a sensitive and reproducible technique for the assessment of LA function and LA mechanical dispersion. Recently, GLS by two‐dimensional speckle‐tracking echocardiography (2D‐STE) has been used for the assessment of regional and global LA function. Therefore, in this study, we aimed to assess LA function and LA mechanical dispersion in breast cancer patients after chemotherapy by echocardiography.

## 2 METHODS

### 2.1 Study population

This is a retrospective study with an initial sample of 100 patients with a histopathologically confirmed diagnosis of breast cancer at an early or locally advanced stage (Stage I–IIIC) between January 2016 and December 2019 at our institution. All patients were female, with a mean age of years (52.8 ± 9.8 years). The exclusion criteria were (1) prior history of chemotherapy, hormone treatment, or radiation; (2) LVEF < 50% before chemotherapy; (3) a previous history of heart failure (HF) and/or coronary heart disease, more than mild valve disease, arrhythmia (AF, atrial flutter, frequent ventricular/atrial premature beat, etc.) and/or cardiomyopathy; and (4) age <20 years or >80 years. All patients underwent chemotherapy one month after modified radical mastectomy. Hematological examination and echocardiography were performed in all patients who received follow‐up after hospital discharge. Overall, 41 (45%) patients received epirubicin (360 mg/m^2^) with concurrent cyclophosphamide, followed by docetaxel (EC‐D); 22 (24%) received trastuzumab with docetaxel and either cyclophosphamide or carboplatin (TCH/TCbH); and 28 (31%) received epirubicin (360 mg/m^2^) with concurrent cyclophosphamide, followed by trastuzumab and docetaxel (EC‐DH). Radiation treatment occurred at a median of 5 months after the operation. CTRCD was defined by a reduction of 10% points in LVEF to a value below 50% (lower limit of normal) or by a relative percentage reduction of more than 15% of LVGLS from baseline.^5^ All patients signed an informed consent. The Dalian Medical University Ethics Committee approved this protocol.

### 2.2 2D‐STE

Echocardiographic examination was performed before chemotherapy (T0), after approximately 6 months of chemotherapy (T6) and after 12 months of chemotherapy (T12) in our department using a Vivid E9 echocardiography system with an M5S transducer (1.7–3.3 MHz) (GE Vingmed Ultrasound), according to the current guidelines and diagnostic criteria by American Society of Echocardiography and European Association of Cardiovascular Imaging.^9^ Continuous ECG recording was performed during the examination. Measurements were made offline on a dedicated workstation (EchoPAC version 202; GE Vingmed Ultrasound). Conventional LV diameter, volume, and function parameters as well as LA parameters were measured. LA volumes (LAVs) were obtained from apical four‐chamber and two‐chamber views through the modified Simpson disc method. Maximum atrial volume (LAVmax), minimal atrial volume (LAVmin), and precontraction atrial volume (LAVp) were calculated. The LAVs were indexed by the body surface area (BSA). The maximum left atrial volume index (LAVImax), minimal left atrial volume index (LAVImin), and precontraction left atrial volume index (LAVIp) were obtained. The LA total ejection fraction (LATEF), the LA expansion index (LAEI), the LA passive ejection fraction (LAPEF) and the LA active ejection fraction (LAAEF) were assessed according to a consensus document of the EACVI/ASE/Industry Task Force.^10^

### 2.3 2D‐STE

Two‐dimensional grayscale images from the apical four‐ and two‐chamber views were acquired at frame rates between 60 and 80 frames/s. Five consecutive heartbeats were digitally stored in cine‐loop format. Measurements were made offline on a dedicated workstation (EchoPAC version 202; GE Vingmed Ultrasound). The LA endocardium was manually traced to avoid including regions of the ostium of the LAA or the lung veins, and the epicardium was automatically traced by the software in the four‐ and two‐chamber apical views. The LA was divided into six segments in each view and then the region of interest was adjusted to match with LA thickness. Segments in which tracking was inadequate were excluded from the analysis despite manual adjustment. If more than three segments were excluded, the subject was removed from the study.

The strain curves of the global and regional LA wall were automatically generated by the software, and the reference point for image analysis was taken at the onset of the QRS complex.^10^ There are two peaks that correspond to LA reservoir function (first peak—LASr) and LA contractile function (second peak—LASct). The difference between LASr and LASct reflects LA conduit function (LAScd). The change of LA mechanical function was expressed as Δ. Δ = the values at T12 − the values at T0. Strain measurements were made by a single operator, and additional reproducibility analysis was performed. LA strain parameters were calculated by averaging all the values obtained in the four‐ and two‐chamber apical views. Absolute values were used for comparison.

LA mechanical dispersion was used to quantify LA mechanical motion synchronization. LA mechanical dispersion was defined as the standard deviation of time to peak positive strain corrected by the R‐R interval (SD‐TPS), and the SD‐TPS values of the LA during reservoir and contraction phases are expressed as SD‐TPSr and SD‐TPSct, indicating the synchronization of LA diastole and contraction.^11^

### 2.4 Intra‐ and interobserver variability

Both intra‐ and interobserver reproducibility were assessed by calculating the difference between the LA strain values of 20 randomly selected patients measured by one observer twice and by a second observer within 48 h.

### 2.5 Statistical analysis

All statistical analyses were carried out using SPSS version 22.0 (SPSS Inc.). Continuous variables are presented as the mean standard deviation (SD), and categorical variables are presented as percentages. The normal distribution of included variables was confirmed by the Kolmogorov–Smirnov test. Analysis of variance of repeated measurement data was used for the comparison of continuous variables among groups. The factors with *p* < .10 were selected by univariate linear regression analysis and included in multivariate regression analysis to identify the independent factors associated with LA function and mechanical dispersion. Inter‐ and intraobserver reproducibilities were assessed using intraclass correlation coefficients to establish and quantify the reproducibility of strain analysis. A *p* < .05 was considered statistically significant.

## 3 RESULTS

### 3.1 Baseline characteristics of the patients

Nine patients (9%) were excluded from the analysis because of poor images that could not be used for strain analysis. A total of 91 female patients with breast cancer were included and followed for one year. The baseline characteristics of the patients are presented in Table S1. The time interval between T0 and T6 was 5.09 ± 1.66 months, and T12 was performed 6.05 ± 2.63 months after the second exam. One chemotherapy plan took 21 days as one cycle. The mean duration of chemotherapy was 6.57 ± 1.9 cycles. The median time elapsed from the last chemotherapy to T12 was 190 ± 35days. None of the patients had developed cardiac complications, including AF.

### 3.2 LV structure and function

**Figure 1:**
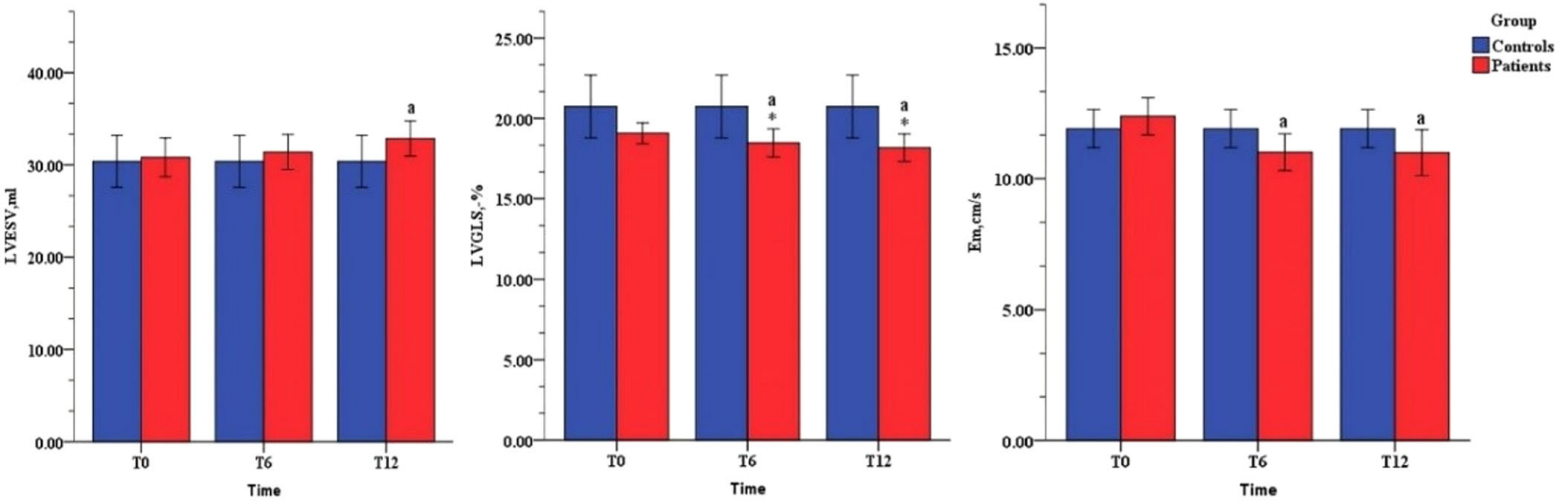
Left ventricle (LV) parameters in the study population. Em, early diastolic lateral mitral annular tissue doppler velocity; LVESV, left ventricular end systolic volume; LVGLS, global longitudinal strain of left ventricle. a, compared with T0, *p* < .05; *, compared with controls, *p* < .05

The trends of echocardiography parameters of LV structure and function are presented in Figure 1. Compared with T0, the LV end systolic volume (LVESV) (T12 32.3 ± 6.9 ml vs. T0 30.7 ± 7.8 ml, *p* < .01) increased significantly at T12, but there was no significant difference compared to controls. LV systolic function was assessed by LVEF and LVGLS. Although a slight decline was observed in LVEF, no significant change was observed. None of the patients developed CTRCD by LVEF. However, 22% of patients developed subclinical CTRCD by LVGLS. The absolute values of LVGLS decreased markedly at T6 and T12 (T0: −20.5% ± 2.3% vs. T6: −18.7% ± 3.0%, *p* < .05; T0: −20.5% ± 2.3% vs. T12: −18.2% ± 3.0%, *p* < .05) and were lower than those of the controls. However, there was no significant difference between T6 and T12. LV diastolic function was evaluated by mitral E, mitral A, E/A ratio, mitral Em, and E/Em ratio. Only Em (T0: 12.4 ± 3.4 cm/s vs. T6: 11.0 ± 3.1 cm/s, *p* < .05, T0: 12.4 ± 3.4 cm/s vs. T12: 11.0 ± 2.4 cm/s, *p* < .05) decreased significantly during follow‐up (Table S2).

### 3.3 LA structure and function

**Figure 2:**
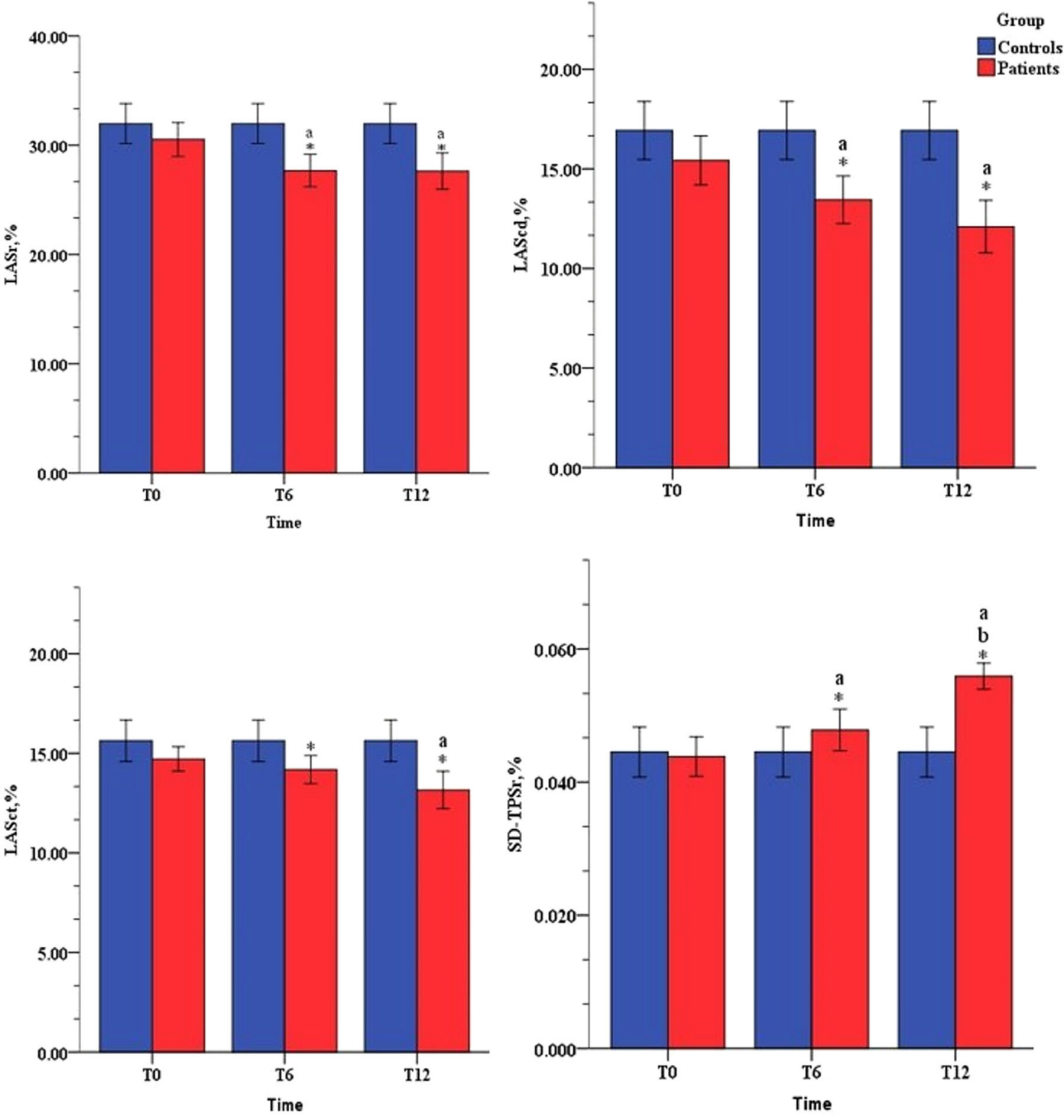
Left atrial (LA) strain parameters for LA function evaluation. LAScd, left atrial strain during conduit phase; LASct, left atrial systolic strain; LASr, left atrial strain during reservoir phase; SD‐TPSr, SD‐TPSct, left atrial mechanical dispersion, the time to peak LASr and LASct corrected by the R‐R interval. a, compared with T0, *p* < .05; b, compared with T6, *p* < .05; *, compared with controls, *p* < .05

No significant change was observed in LA structural parameters or phasic function determined by the volumetric method (Table S3). STE parameters deteriorated significantly after chemotherapy and were obviously lower than those of controls (Figures 2 and S1A,B). LASr (T0: 30.9% ± 6.4% vs. T6: 28.9% ± 6.3%, *p* < .05; T0: 30.9% ± 6.4% vs. T12: 28.6% ± 6.0%, *p* < .05) declined over time. LAScd (T0: 15.8% ± 5.5% vs. T6: 14.4% ± 5.1%, *p* < .05; T0: 15.8% ± 5.5% vs. T12: 14.3% ± 4.5%, *p* < .05) declined significantly, but no obvious change was observed between T6 and T12. LASct (T0: 14.9% ± 2.9% vs. T12: 14.3% ± 3.0%, *p* < .05) declined markedly at T12 (Table S4).

### 3.4 Changes in LA mechanical dispersion over time

**Figure 3:**
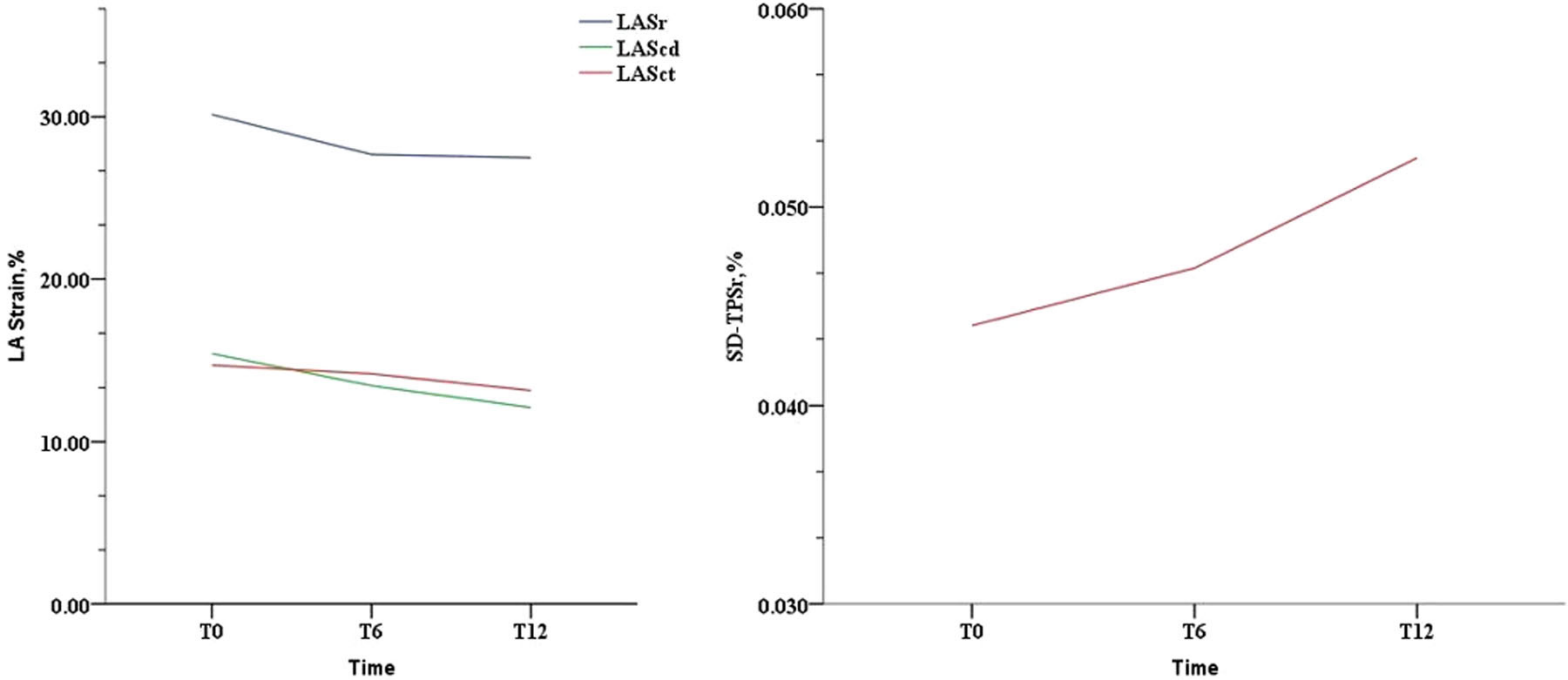
Changes in left atrial (LA) function and mechanical dispersion over time. LAScd, left atrial strain during conduit phase; LASct, left atrial systolic strain; LASr, left atrial strain during reservoir phase; SD‐TPSr, left atrial mechanical dispersion, the time to peak LASr corrected by the R‐R interval

The LA mechanical dispersion parameter SD‐TPSr increased from T6 and persisted with time (T0: 4.5% ± 1.4% vs. T6: 5.2% ± 1.9%, *p* < .05; T0: 4.5% ± 1.4% vs. T12: 6.1% ± 1.1%, *p* < .05). However, SD‐TPSct showed no significant changes over time (Table S4). The inter‐ and intraobserver variabilities for LA strain showed fairly consistent repeatability (Table S5). Changes in LA mechanical dispersion over the one‐year period are graphically depicted in Figure 3. SD‐TPSr showed a sustained increase during the follow‐up. The trend was more obvious at T6.

### 3.5 Univariate and multivariate linear regression analyses

After univariate analysis of clinical confounders, all measures of cardiac structure and function, the factors with *p *< .10 were included in multivariate regression analysis (Table 1). After adjustment, LAVIp (0.399, 95% CI: 0.610, 1.756, *p* = .000) was independently associated with ΔLASr, LAPEF (−0.325, 95% CI: −45.123, −10.676, *p* = .002) and age (0.227, 95% CI: 0.021, 0.350, *p* = .027) were independently associated with ΔLAScd, and LAVImax (0.341, 95% CI: 0.192, 0.723, *p* = .001) was independently associated with ΔLASct (Table 1). LAVImax (0.505, 95%: CI 0.000, 0.001, *p* = .039) and mitral E (−0.256, 95% CI: 0.000, 0.000, *p* = .024) were independently associated with ΔLA SD‐TPSr (Table 2).

**Table 1:** Multivariate linear regression analysis of subject characteristics influencing ΔLASr, ΔLAScd, and ΔLASct

Dependent variable	Univariate		Multivariate		
Independent variables	95% CI lower‐upper	*p*	Standardized beta	95% CI lower‐upper	*p*
ΔLASr					
Age	0.037, −0.615	.028	‐	‐	‐
Heart rate	−0.668, −0.177	.040	‐	‐	‐
LAVImax	0.364, 1.381	−.001	‐	‐	‐
LAVImin	0.666, 2.703	−.001	‐	‐	‐
LAVIp	0.610, 1.756	.000	0.399	0.610, 1.756	.000
LAPEF	−80.159, −21.676	.001	‐	‐	‐
LAEI	−10.220, 0.465	.073	‐	‐	‐
ΔLAScd					
Age	0.114, 0.439	.001	0.227	0.021, 0.350	.027
Heart rate	−0.339, 0.047	.010	‐	‐	
LAVImax	0.114, 0.716	.007	‐	‐	‐
LAVImin	0.343, 1.531	.002	‐	‐	
LAVIp	0.319, 0.990	.000	‐	‐	
LAPEF	−51.100, −18.010	.000	−0.325	−45.123, −10.676	.002
LATEF	−50.244, −1.760	.036	‐	‐	
LAEI	−6.569, −0.429,	.026	‐	‐	
Mitral E	−0.210, −0.004	.04	‐	‐	
Mitral E/A	−14.012, −1.829	.011	‐	‐	
Mitral Em	−1.169, −0.211	.005	‐	‐	
ΔLASct					
Chemotherapy duration	−1.687, −0.113	.025	‐	‐	‐
Elapsed time	0.005, 0.090	.029	‐	‐	‐
Heart rate	−0.357, −0.102	.001	‐	‐	‐
LAVImax	0.192, 0.723	.001	0.341	0.192, 0.723	.01
LAVImin	0.206, 1.289,	.007	‐	‐	‐
LAVIp	0.221, 0.836	.001	‐	‐	‐
LAPEF	−32.284, −0.442	.044	‐	‐	‐

Abbreviations: CI, confidence interval; Elapsed time, the median time elapsed from the last chemotherapy to T12; LAEI, left atrial expansion index; LAPEF, left atrial passive emptying fraction; LAScd, left atrial strain during conduit phase; LASct, left atrial systolic strain; LASr, left atrial strain during reservoir phase; LATEF, left atrial total emptying fraction; LAVImax, maximum left atrial volume index; LAVImin, minimum left atrial volume index; LAVIp, precontraction left atrial volume index; mitral A, late diastolic mitral flow; mitral E, early diastolic mitral flow; mitral Em, early diastolic lateral mitral annular tissue doppler velocity.

**Table 2:** Multivariate linear regression analysis of subject characteristics influencing of ΔSD‐TPSr

Dependent variable: ΔSD‐TPSr	Univariate		Multivariate		
Independent variables	95% CI lower‐upper	*p*	Standardized beta	95% CI lower‐upper	*p*
LAVImax	0.000, 0.000	.025	0.505	–0.001, 0.000	.039
LAVIp	0.000, 0.000	.061	‐	‐	‐
LASr	0.000, 0.000	.010	‐	‐	‐
LAScd	0.000, 0.000	.013	‐	‐	‐
Mitral E	0.000, 0.000	.004	−0.256	0.000, 0.000	.024
Mitral Em	−0.001, 0.000	.025	‐	‐	‐

Abbreviations: CI, confidence interval; LAScd, left atrial strain during conduit phase; LASr, left atrial strain during reservoir phase; LAVImax, maximum left atrial volume index; LAVIp, precontraction left atrial volume index; mitral E, early diastolic mitral flow; mitral Em, early diastolic lateral mitral annular tissue doppler velocity; SD‐TPSr, left atrial mechanical dispersion, the time to peak LASr corrected by the R‐R interval.

## 4 DISCUSSION

There were several findings in this study that deserve further discussion. (1) After chemotherapy, the LA strain parameters decreased significantly in breast cancer patients and were lower than those of the controls. In addition, LASr and LAScd (at T6) decreased earlier than LASct did (at T12). (2) The standard deviation of time to peak LASr corrected by the R‐R interval (SD‐TPSr), an index reflecting the synchronization of atrial mechanical function, increased significantly in the follow‐up. (3) Except for LAVIp, there were no significant changes in LAVI or LA functions based on volume, such as LATEF and LAEI. Thus, LA strain can detect atrial dysfunction earlier than LAV parameters. (4) Regarding LV, LVESV was augmented at T12. LVGLS declined significantly and was lower than controls from T6. However, no significant change was observed in LVEF. The parameters of diastolic function‐Em decreased markedly at T12.

### 4.1 LAV

LAV remains the crucial echocardiographic parameter to assess remodeling and indirectly the function of the LA and is a practical prognostic tool.^12^ However, controversies remain regarding the change in LAV after chemotherapy. Yaylali et al. showed that compared with controls, LAVImax and LAVIp increased significantly after chemotherapy.^13^ In breast cancer patients receiving chemotherapy, LA dilatation has been proven to be related to the occurrence of cardiac dysfunction.^14^ However, in a study conducted among long‐term survivors of childhood cancer treated with anthracyclines, the LAVI of the chemotherapy group decreased notably compared with that of the control group. However, LAEF did not change significantly. The author explained that this might be due to fibrosis and cardiac remodeling.^15^ Another study showed that among children exposed to anthracyclines, the short‐term effects on LA function were small for patients with preserved LVEF.^16^ In our study, the LAV of breast cancer patients increased after chemotherapy, but there was no significant difference. The shorter follow‐up time, the younger subjects, the smaller sample sizes, vomiting or inadequate intake due to chemotherapy, and different measurement methods might be the main reasons for the inconsistency with the studies above.

### 4.2 LA strain

LA modulates LV filling pressure and cardiovascular performance by functioning as a reservoir, conduit, and booster pump, which plays an integral pathophysiological role in LV diastolic dysfunction. Atrial strain showed a good correlation with pulmonary capillary wedge pressure, even better than the E/e' ratio in advanced HF.^17^ Theoretically, a reduction in LA performance mirrors diastolic dysfunction.

Atrial strain has been evaluated in multiple conditions, such as hypertension, diabetes, AF, HF, ischemic and valvular heart disease, and has been included for the assessment of prognostic implications.^9^ In our study, we found that LA strain could detect atrial dysfunction earlier than the parameters of volume. Substantial reductions were observed in all the parameters of LA strain in this study. Li et al. found that compared with the control group, the strain parameters of LA function decreased significantly among long‐term survivors of childhood cancer chemotherapy, but there was no significant difference in LAV assessment.^15^ The results were in close agreement with our study. However, Timóteo et al. found that only LASct declined during the first year of breast cancer treatment, and no significant changes in LASr and LAScd were observed in their study.^6^ The results above are not consistent with our study, and the main reasons may include the different baseline characteristics, the different treatment regimens and dosages, and the different exam times. In our study, LASr and LAScd declined earlier than LASct, which was consistent with other studies. Shi et al. found that LAScd was reduced and LASct was increased immediately after completion of anthracycline therapy in non‐Hodgkin lymphoma patients.^18^ A possible reason might be that impaired LV relaxation reduced passive atrial conduit function (LAScd) and LA stiffness decreased reservoir function (LASr) due to chemotherapy. In the early stages, LA contraction (LASct) is augmented as a compensatory mechanism, but with prolonged dysfunction, LASct decreases due to LA dilation and stiffness.

LAVIp, LAPEF, and LAVImax were independently associated with ΔLA strain in this study, which suggested that the impairment of LA mechanical function and synchronization assessed by 2D‐STE was significantly related to the change of LAV, which indicated the increase of LA pressure.

### 4.3 LA dispersion and arrhythmia

Chemotherapy is a frequent cause of arrhythmias including AF.^19^ Several anticancer agents were found to induce AF, with a reported incidence for anthracycline of 2%–10%^20^, ^21^ in breast cancer patients treated with trastuzumab. Therefore, it is necessary to monitor the risk factors related to arrhythmias during chemotherapy. It has been reported that after chemotherapy, the electromechanical delay of LA measured by tissue Doppler imaging was significantly prolonged, which may be associated with the development of arrhythmias, especially AF.^13^ LA SD‐TPSr is a novel parameter reflecting mechanical dispersion. Kawakami et al. pointed out that LA mechanical dispersion obtained from strain echocardiography might provide incremental information in the prediction of new‐onset AF beyond the traditional parameters in a general population.^22^ LA mechanical dispersion has been shown to be increased in patients with nonrheumatic paroxysmal AF.^23^ In our study, SD‐TPSr showed a sustained increase during the follow‐up, and the trend was more obvious after T6. ΔSD‐TPSr was independently associated with LAVImax and mitral E in this study, which suggested that the change of SD‐TPSr might be associated with the LV diastolic function. Moreover, the results of this study showed that the decline in LA function was accompanied by an increase in LA mechanical dispersion in patients after chemotherapy, and the clinical significance of these phenomena still needs further study. The effects of chemotherapy on atrial myocardium fibers over time remain unclear, though they may result in autonomic nervous dysfunction through oxidative stress, decreases in intracardiac conduction and a heterogeneous dispersion of repolarization, leading to the dyssynchrony of mechanical movement of the atrium.^24^

### 4.4 Limitations

There are several limitations in the present study. The sample size was relatively small. No subgroup analysis was conducted according to the chemotherapy regimen. The follow‐up period was only up to one year. The relationship between atrial parameters and possible arrhythmia was not monitored, and paroxysmal arrhythmias may have been missed. We used software designed for LV strain analysis to obtain LA strain because of the lack of dedicated atrial software. Vendor differences arising from differences between edge tracking and speckle tracking may affect the reliability of LA strain and SD‐TPS. Further prospective studies will be required to determine the clinical significance of our observed findings.

## 5 CONCLUSION

The mechanical function of LA changed after chemotherapy in breast cancer patients. The decrease in functional indicators measured by 2D‐STE occurred before the changes in LAV parameters. With the decrease of LA mechanical function, LA mechanical dispersion assessed by 2D‐STE increased significantly, and its clinical value needs to be further studied.

## CONFLICTS OF INTEREST

The authors declare no conflicts of interest.

## Supporting information

Supplemental Figure S1. Serial STE in one patient. A. LA strain measurement before chemotherapy showed an LASr of 38.81%, SD‐TPSr was 3.6%.

B. LA strain measurement after 14 months chemotherapy showed an LASr of 27%, SD‐TPSr was 5.4%. STE, speckle tracking echocardiography; LASr, left atrial strain during reservoir phase; SD‐TPSr, the time to peak LASr corrected by the R‐R interval during reservoir phase.

Supporting information.

Supporting information.

Supporting information.

Supporting information.

Supporting information.

## Data Availability

The data used to support the findings of this study are available from the corresponding author upon request.

## References

[1] Cancer Stat Facts: Female Breast Cancer. Surveillance, Epidemiology, and End Results Program. Accessed June 17, 2017. https://seer.cancer.gov/statfacts/html/breast.html

[2] Seidman A , Hudis C , Pierri MK , et al. Cardiac dysfunction in the trastuzumab clinical trials experience. J Clin Oncol. 2002;20:1215‐1221. 11870163 10.1200/JCO.2002.20.5.1215

[3] Cardinale D , Colombo A , Bacchiani G , et al. Early detection of anthracycline cardiotoxicity and improvement with heart failure therapy. Circulation. 2015;131:1981‐1988. 25948538 10.1161/CIRCULATIONAHA.114.013777

[4] Plana JC , Galderisi M , Barac A , et al. Expert consensus for multimodality imaging evaluation of adult patients during and after cancer therapy: a report from the American Society of Echocardiography and the European Association of Cardiovascular Imaging. Eur Heart J Cardiovasc Imaging. 2014;15(10):1063‐1093. 25239940 10.1093/ehjci/jeu192 PMC4402366

[5] Zamorano JL , Lancellotti P , Rodriguez Muñoz D , et al. 2016 ESC position paper on cancer treatments and cardiovascular toxicity developed under the auspices of the ESC committee for practice guidelines. Eur Heart J. 2016;37:2768‐2801. 27567406 10.1093/eurheartj/ehw211

[6] Timoteo AT , Moura Branco L , Filipe F , et al. Cardiotoxicity in breast cancer treatment: what about left ventricular diastolic function and left atrial function? Echocardiography. 2019;36(10):1806‐1813. 31573712 10.1111/echo.14487

[7] Park H , Kim KH , Kim HY , et al. Left atrial longitudinal strain as a predictor of cancer therapeutics‐related cardiac dysfunction in patients with breast cancer. Cardiovasc Ultrasound. 2020;18:28. 32693802 10.1186/s12947-020-00210-5 PMC7374848

[8] Cho GY , Jo SH , Kim MK , et al. Left atrial dys‐synchrony assessed by strain imaging in predicting future development of atrial fibrillation in patients with heart failure. Int J Cardiol. 2009;134:336‐341. 18804876 10.1016/j.ijcard.2008.08.019

[9] Lang RM , Badano LP , Mor‐Avi V , et al. Recommendations for cardiac chamber quantification by echocardiography in adults: an update from the American society of echocardiography and the European association of cardiovascular imaging. J Am Soc Echocardiogr. 2015;28:1‐39. 25559473 10.1016/j.echo.2014.10.003

[10] Badano LP , Kolias TJ , Muraru D , et al. Standardization of left atrial, right ventricular, and right atrial deformation imaging using two‐dimensional speckle tracking echocardiography: a consensus document of the EACVI/ASD/Industry Task Force to standardize deformation imaging. Eur Heart J Cardiovasc Imaging. 2018;19:591‐600. 29596561 10.1093/ehjci/jey042

[11] Shang Z , Su D , Cong T , et al. Assessment of left atrial mechanical function and synchrony in paroxysmal atrial fibrillation with two‐dimensional speckle tracking echocardiography. Echocardiography. 2017;34:176‐183. 28240425 10.1111/echo.13434

[12] Pathan F , D' Elia N , Nolan MT , Marwick TH , Negishi K . Normal ranges of left atrial strain by speckle‐tracking echocardiography: a systematic review and meta‐analysis. J Am Soc Echocardiogr. 2017;30:59‐70. 28341032 10.1016/j.echo.2016.09.007

[13] Yaylali YT , Saricopur A , Yurtdas M , Senol H , Gokoz‐Dogu G . Atrial function in patients with breast cancer after treatment with Anthracyclines. Arq Bras Cardiol. 2016;107(5):411‐419. 27812678 10.5935/abc.20160146 PMC5137385

[14] Bergamini C , Dolci G , Rossi A , et al. Left atrial volume in patients with HER2‐positive breast cancer: one step further to predict trastuzumab-related cardiotoxicity. Clin Cardiol. 2018;41(3):349‐353. 29569424 10.1002/clc.22872 PMC6490137

[15] Li VW , Lai CT , Liu AP , et al. Left atrial mechanics and integrated calibrated backscatter in anthracycline‐treated long‐term survivors of childhood cancers. Ultrasound Med Biol. 2017;43(9):1897‐1905. 28645798 10.1016/j.ultrasmedbio.2017.05.017

[16] Patel NR , Chyu CK , Satou GM , Halnon NJ , Nguyen KL . Left atrial function in children and young adult cancer survivors treated with anthracyclines. Echocardiography. 2018;35(10):1649‐1656. 30053329 10.1111/echo.14100

[17] Cameli M , Lisi M , Mondillo S , et al. Left atrial longitudinal strain by speckle tracking echocardiography correlates well with left ventricular filling pressures in patients with heart failure. Cardiovasc Ultrasound. 2010; 8:14. 20409332 10.1186/1476-7120-8-14 PMC2868789

[18] Shi J , Guo Y , Cheng L , Song F , Shu X . Early change in left atrial function in patients treated with anthracyclines assessed by real‐time three‐dimensional echocardiography. Sci Rep. 2016;6(1):25512. 27149058 10.1038/srep25512 PMC4857739

[19] Erichsen R , Christiansen CF , Frøslev T , Jacobsen J , Sørensen HT . Intravenous bisphosphonate therapy and atrial fibrillation/flutter risk incancer patients: a nationwide cohort study. Br J Cancer. 2011;105:881‐883. 21878939 10.1038/bjc.2011.338 PMC3185951

[20] Guglin M , Aljayeh M , Saiyad S , et al. Introducing a new entity: chemotherapy‐induced arrhythmia. Europace. 2009;11(12):1579‐1586. 19801562 10.1093/europace/eup300

[21] Yuan M , Tse G , Zhang Z , et al. The incidence of atrial fibrillation with trastuzumab treatment: a systematic review and meta‐analysis. Cardiovasc Ther. 2018;36(6):e12475. 30372591 10.1111/1755-5922.12475

[22] Kawakami H , Ramkumar S , Nolan M , et al. Left atrial mechanical dispersion assessed by strain echocardiography asan independent predictor of new‐onset atrial fibrillation: a case‐control study. J Am Soc Echocardiogr. 2019;32:1268‐1276. 31466848 10.1016/j.echo.2019.06.002

[23] Aya H , Yoshihiro K , Makoto W . Echocardiographic parameters and the risk of incident atrial fibrillation: the Suita study. J Epidemiol. 2020;30(4):183‐187. 30930375 10.2188/jea.JE20180251 PMC7064552

[24] Aktoz M , Yilmaztepe M , Tatli E , et al. Assessment of ventricular and left atrial mechanical functions, atrial electromechanical delay e P wave dispersion in patients with scleroderma. Cardiol J. 2011;18(3):261‐269. 21660915

