# Supplementary figures and images for: Evaluation of left atrial function and mechanical dispersion in breast cancer patients after chemotherapy
Source: Clin Cardiol. 2022 Mar 16;45(5):540–8. doi: 10.1002/clc.23813 (PMC9045082; doi:10.1002/clc.23813)

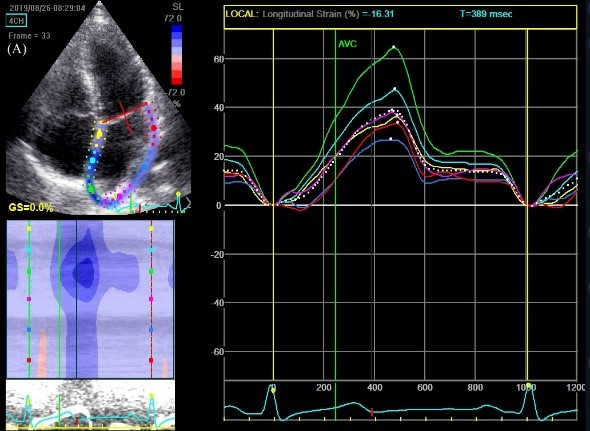

Supplement: Supplementary file 1 — Supplemental Figure S1. Serial STE in one patient. A. LA strain measurement before chemotherapy showed an LASr of 38.81%, SD‐TPSr was 3.6%. [file CLC-45-540-s005.tif]

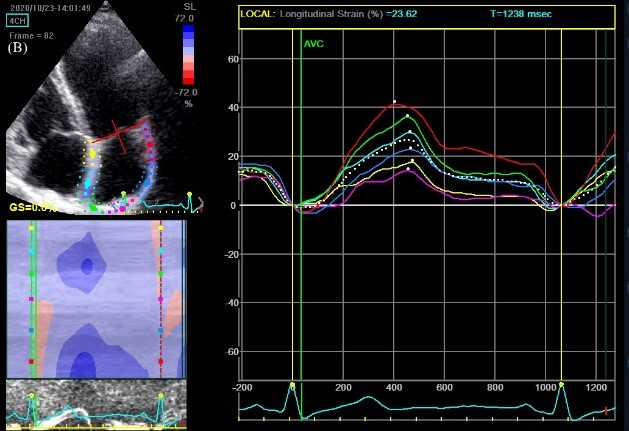

Supplement: Supplementary file 2 — B. LA strain measurement after 14 months chemotherapy showed an LASr of 27%, SD‐TPSr was 5.4%. STE, speckle tracking echocardiography; LASr, left atrial strain during reservoir phase; SD‐TPSr, the time to peak LASr corrected by the R‐R interval during reservoir phase. [file CLC-45-540-s006.tif]
