# Supplementary material for: Evaluation of left atrial function and mechanical dispersion in breast cancer patients after chemotherapy
Source: Clin Cardiol. 2022 Mar 16;45(5):540–8. doi: 10.1002/clc.23813 (PMC9045082; doi:10.1002/clc.23813)
Supplement: Supplementary file 3 — Supporting information. [file CLC-45-540-s007.docx]

Supplemental TableS1. Baseline characteristics of the subjects

| Variables | Patients  （N=91） | Controls  (n=30) | P |
| --- | --- | --- | --- |
| Age (year) | 52.8±9.8 | 52.1±8.0 | 0.735 |
| Heart rate (beats/min) | 76.7±11.2 | 73.0±5.9 | 0.024 |
| BMI (kg/m^2^) | 24.6±3.6 | 24.4±2.8 | 0.778 |
| BSA (m^2^) | 1.69±0.11 | 1.67±0.13 | 0.427 |
| Hypertension (%) | 12(13) | 4(13) | 1.000 |
| Diabetes Mellitus (%) | 6(7) | 4(13) | 0.435 |
| ACEI or ARB, n (%) | 12(13) | 2(7) | 0.523 |
| Beta blocker, n (%) | 6(7) | 2(7) | 1.000 |
| Chemotherapy (%) |  |  |  |
| Epirubicin + Cyclophosphamide+ Docetaxel  Trastuzumab+Docetaxel+Cyclophosphamide/Carboplatin  Epirubicin+Cyclophosphamide+Anti-HER2+Docetaxel | 41(45)  22 (24)  28 (31) |  |  |
| Radiotherapy (%) | 77(85) |  |  |

BMI, body mass index; BSA, body surface area; ACEI, angiotensin-converting enzyme inhibitors; ARB, angiotensin receptor blockers.
