# Supplementary material for: Evaluation of left atrial function and mechanical dispersion in breast cancer patients after chemotherapy
Source: Clin Cardiol. 2022 Mar 16;45(5):540–8. doi: 10.1002/clc.23813 (PMC9045082; doi:10.1002/clc.23813)
Supplement: Supplementary file 4 — Supporting information. [file CLC-45-540-s003.docx]

| Variables | Groups | T0 | T6 | T12 | P |
| --- | --- | --- | --- | --- | --- |
| LVEDV (ml) | patients | 79.0±14.1 | 79.7±15.1 | 81.4±14.4 | 0.347 |
|  | controls | 84.7±15.9 |  |  |  |
| LVESV (ml) | patients | 30.7±7.8 | 30.4±8.3 | 32.3±6.9 ^a^ | 0.005 |
|  | controls | 30.4±7.5 |  |  |  |
| LVEF（%） | patients | 66.3±5.1 | 65.5±5.0 | 64.9±4.1 | 0.172 |
|  | controls | 64.8±3.4 |  |  |  |
| LVGLS (%) | patients | -20.5±2.3 | -18.7±3.0 ^a,*^ | -18.2±3.0 ^a,*^ | 0.000 |
|  | controls | 20.7±3.2 |  |  |  |
| Mitral E (cm/s) | patients | 77.1±16.1 | 75.4±16.8 | 74.5±16.7 | 0.405 |
|  | controls | 77.5±9.8 |  |  |  |
| Mitral A (cm/s) | patients | 77.1±14.4 | 78.2±16.5 | 74.3±16.7 | 0.524 |
|  | controls | 73.6±15.3 |  |  |  |
| Mitral E/A | patients | 1.02±0.27 | 1.00±0.26 | 1.02±0.21 | 0.273 |
|  | controls | 1.09±0.21 |  |  |  |
| Mitral Em(cm/s) | patients | 12.4±3.4 | 11.0±3.1^a^ | 11.0±2.4^a^ | 0.004 |
|  | controls | 11.9±2.0 |  |  |  |
| Mitral E/Em | patients | 6.6±2.0 | 7.2±2.1 | 7.0±2.0 | 0.205 |
|  | controls | 6.7±1.3 |  |  |  |

Supplemental Table S2. Left Ventricular Structure and Function

a, compared with T0, P<0.05; *, compared with controls, P<0.05. LVEDV, left ventricular end diastolic volume; LVESV, left ventricular end systolic volume; LVEF, left ventricular ejection fraction; LVGLS, global longitudinal strain of left ventricle; Mitral E, early diastolic mitral flow; Mitral A, late diastolic mitral flow; Mitral Em, early diastolic lateral mitral annular tissue doppler velocity.
