# Supplementary material for: Evaluation of left atrial function and mechanical dispersion in breast cancer patients after chemotherapy
Source: Clin Cardiol. 2022 Mar 16;45(5):540–8. doi: 10.1002/clc.23813 (PMC9045082; doi:10.1002/clc.23813)
Supplement: Supplementary file 5 — Supporting information. [file CLC-45-540-s001.docx]

Supplemental Table S3. LA phasic function determined by volumetric method in the study population

| Variable | Groups | T0 | T6 | T12 | P |
| --- | --- | --- | --- | --- | --- |
| LAVImax (ml/m^2^) | patients | 21.9±5.4 | 22.4±5.5 | 24.2±5.5 | 0.103 |
|  | controls | 22.2±3.5 |  |  |  |
| LAVImin (ml/m^2^) | patients | 7.8±2.7 | 8.2±3.0 | 8.9±3.1 | 0.129 |
|  | controls | 8.6±2.3 |  |  |  |
| LAVIp (ml/m^2^) | patients | 14.4±4.7 | 15.2±4.7 | 16.5±4.6^*^ | 0.277 |
|  | controls | 14.3±3.1 |  |  |  |
| LATEF (%) | patients | 64.5±6.8 | 64.1±6.7 | 62.8±6.7 | 0.526 |
|  | controls | 60.7±10.8 |  |  |  |
| LAEI (%) | patients | 191.1±53.8 | 187.8±55.6 | 176.6±50.9 | 0.497 |
|  | controls | 170.1±61.0 |  |  |  |
| LAPEF (%) | patients | 34.9±9.4 | 32.4±8.8 | 32.8±8.0 | 0.627 |
|  | controls | 35.1±13.9 |  |  |  |
| LAAEF (%) | patients | 448±10.5 | 46.4±9.4 | 44.3±8.7 | 0.289 |
|  | controls | 44.4±11.6 |  |  |  |

*，compared with controls, P<0.05. LAVImax, maximum left atrial volume index; LAVImin, minimum left atrial volume index; LAVIp, precontraction left atrial volume index; LATEF, left atrial total emptying fraction; LAEI, left atrial expansion index; LAPEF, left atrial passive emptying fraction; LAAEF, left atrial active emptying fraction.
