# Supplementary material for: Evaluation of left atrial function and mechanical dispersion in breast cancer patients after chemotherapy
Source: Clin Cardiol. 2022 Mar 16;45(5):540–8. doi: 10.1002/clc.23813 (PMC9045082; doi:10.1002/clc.23813)
Supplement: Supplementary file 6 — Supporting information. [file CLC-45-540-s004.docx]

Supplemental Table S4. LA phasic function determined by strain method in the study population

| Variable | Groups | T0 | T6 | T12 | P |
| --- | --- | --- | --- | --- | --- |
| LASr（%） | patients | 31.9±6.4 | 28.9±6.3^a,*^ | 28.6±6.0^a,*^ | 0.000 |
|  | controls | 32.0±4.9 |  |  |  |
| LAScd（%） | patients | 15.8±5.5 | 14.4±5.1^a,*^ | 14.3±4.5^a,*^ | 0.000 |
|  | controls | 16.9±3.9 |  |  |  |
| LASct（%） | patients | 14.9±2.9 | 14.6±3.1^*^ | 14.3±3.0^a,*^ | 0.008 |
|  | controls | 15.6±2.8 |  |  |  |
| SD-TPSr (%) | patients | 4.5±1.4 | 5.2±1.9^a,*^ | 6.1±1.1^a,b,*^ | 0.000 |
|  | controls | 4.5±1.0 |  |  |  |
| SD-TPSct（%） | patients | 4.4±1.6 | 4.3±2.0 | 4.2±1.6 | 0.406 |
|  | controls | 4.1±1.3 |  |  |  |

a, compared with T0, P<0.05; b, compared with T6, P<0.05; *, compared with controls, P<0.05. LASr, left atrial strain during reservoir phase; LAScd, left atrial strain during conduit phase; LASct, left atrial systolic strain; SD-TPSr, SD-TPSct, left atrial mechanical dispersion, the time to peak LASr and LASct corrected by the R-R interval.
