# Supplementary material for: Evaluation of left atrial function and mechanical dispersion in breast cancer patients after chemotherapy
Source: Clin Cardiol. 2022 Mar 16;45(5):540–8. doi: 10.1002/clc.23813 (PMC9045082; doi:10.1002/clc.23813)
Supplement: Supplementary file 7 — Supporting information. [file CLC-45-540-s002.docx]

Supplemental Table S5. Reproducibility of LA strain parameters

|  | Inter-observer | Intra-observer |
| --- | --- | --- |
| Parameters | ICC | ICC |
| LASr | 0.92 | 0.89 |
| LAScd | 0.81 | 0.85 |
| LASct | 0.83 | 0.88 |
| SD-TPSr | 0.93 | 0.96 |
| SD-TPSct | 0.91 | 0.92 |

LASr, left atrial strain during reservoir phase; LAScd, left atrial strain during conduit phase; LASct, left atrial systolic strain; SD-TPSr, SD-TPSct, left atrial mechanical dispersion, the time to peak LASr and LASct corrected by the R-R interval.
